# An In Vitro Comparative Study on the Antimicrobial Effects of Bioglass 45S5 vs. Calcium Hydroxide on Enterococcus Faecalis

**Published:** 2011-02-15

**Authors:** Payman Mehrvarzfar, Hengameh Akhavan, Hossein Rastgarian, Nahid Mohammadzade Akhlagi, Reza Soleymanpour, Anahid Ahmadi

**Affiliations:** 1. Department of Endodontics, Dental School, Islamic Azad University of Medical Science, Tehran, Iran.; 2. Department of Microbiology, Dental School, Islamic Azad University of Medical Science, Tehran, Iran.; 3. Private Practice, Tehran, Iran.

**Keywords:** Bioglass, Calcium Hydroxide, Direct Exposure Test, Root Canal Medicaments

## Abstract

**INTRODUCTION:**

An ideal intracanal medicament should be able to eliminate any remaining intracanal microorganism. The aim of this study was to compare the antimicrobial effects of Bioglass 45S5 with calcium hydroxide on Enterococcus (E) faecalis in-vitro.

**MATERIALS AND METHODS:**

Direct exposure test (DET) was used to evaluate the antimicrobial effect of Bioglass 45S5, calcium hydroxide and normal saline (control group) on 80 paper cones contaminated with E. faecalis suspension. All samples were aseptically transferred into BHI culture medium to quantify microbial concentration in periods of 1, 24, 48 and 72 hours. Turbidity of the culture medium was measured via optical density (OPD) method with a spectrophotometer (wavelength=540nm). Results were then analysed statistically using student t-test.

**RESULTS:**

Mean difference of optical density between Bioglass 45S5 and calcium hydroxide appeared insignificant within 1 hour of the test period (P>0.05); however calcium hydroxide showed significantly greater antimicrobial properties after 24 hours (P<0.05). Antimicrobial effect of both materials displayed significant increases with time.

**CONCLUSION:**

Although both Bioglass 45S5 and calcium hydroxide exhibited antimicrobial effects against E. faecalis, neither attained complete eradication of bacteria. However, calcium hydroxide seemed to have superior disinfecting effect.

## INTRODUCTION

Bacteria and their by-products play a major role in periradicular diseases and its progress; in fact, they are the main interfering factors in the periapical repair process [1][2]. The disinfection of a complicated root canal system is crucial for long term success [3][4]. However, even after chemo-mechanical debridement of the root canals, it is difficult, if not impossible, to achieve complete disinfection [5][6][7].

Recent microbiologic studies demonstrate wide-spread presence of resistant microorganisms such as E. faecalis and Candida Albicans in persistent canal infections and secondary infections in endodontic failures [4][8][9][10]. Appropriate intracanal medicaments between treatment sessions can offer beneficial antimicrobial effects as they are retained in canal space for relatively longer periods; therefore numerous searches have been performed to find the perfect intracanal medicament [3][5][6][10].

An ideal intracanal medicament should have wide antimicrobial properties, high compatibility with periapical tissue and induce hard tissue repair as well as reduce inflammation [3]. Calcium hydroxide is currently known as the gold standard for intracanal medicaments. However, there are doubts about its antimicrobial effect against yeast and Enterococci [1][4][10], specially within dentinal tubules of root canals [1][4][5][6][7][10][11].

Bioglass, a silica based melt-derived glass has had wide uses in medicine and dentistry [12][13]. This active biomaterial has antimicrobial and anti-inflammatory effects, it has the ability to bond to soft and hard tissues; moreover, when implanted in bone, it displays osteoconductive properties which may assist the repair of bony defects [12][13][14][15][16][17]. The wide-spectrum antimicrobial effect of bioactive glass (BAGs) S53P4 on different oral microorganisms has been reported which justifies its use as an intracanal medicament and intraosseous matrix in endodontics [18][19]. Krithikadatta et al. compared disinfecting properties of 0.2% chlorhexidine (CHX), 2% metronidazole and bioactive glass S53P4 with calcium hydroxide on dentinal tubules. All groups demonstrated similar results [4].

Bioglass S53P4 is thought to be the most effective glass on pathogenic microorganisms [16]. Waltimo et al. reported a strong antimicrobial activity for Bioglass 45S5 against Enterococci isolated from persistent canal infections [20]. Despite different existing formulas available for bioactive glass, the 45S5 appears the best as it consists of 45% SiO2, 24.5% CaO, 24.5% Na, 2O.6% P2O5. These particles are not only osteoconductive, but also have a composition similar to bone in regards to calcium and phosphor ions [13][14][15][16][17].

The aim of the current in vitro study was to compare the antimicrobial effect of bioactive glass 45S5 (Nova Bone) and calcium hydroxide on E. faecalis using a direct exposure test (DET).

## MATERIALS AND METHODS

This experimental study comprised of eighty paper cones contaminated with E. faecalis (ATCC 19818). Sterile absorbent points #50 were floated in a 10(8) CFU/mL concentration of E. faecalis suspension for 5 minutes. The paper cones were then regarded as the study samples. All experimental procedures were carried out under strict aseptic conditions. The contaminated paper cones were randomly divided into four test and control groups as outlined below.

Test group 1 (n=20): 5.1 g bioactive glass 45S5 powder (NovaBone, US Biomaterial Corporation, USA/ Percentage weight rate: SIO2 (45%), CaO (24.5%), Na (24.5%), P2O5 (20.6%)) mixed with 4mL sterile distilled water to form a toothpaste consistency paste.

Test group 2 (n=20): 6.2 g calcium hydroxide Ca(OH)_2_ (Merk, Germany) mixed with 12mL sterile distilled water to form a paste with the same consistency as the Bioglass mixture.

The control negative group (n=20) was exposed to normal saline and autoclaved, whereas control positive samples were only exposed to normal saline. Direct exposure test (DET) was used for the evaluation of antimicrobial effects of study samples. Each paper cone was separately placed in a sterile petri plate completely covered with experimental materials or saline (control groups). Plates were placed in a 37°C, 100% humidity incubator. A total of 5 samples were aseptically removed from each group at 1, 24, 48, 72 hours intervals and irrigated with normal saline to completely eliminate materials. Each paper cone was then inserted into sterile lab tubes containing 5mL BHI (brain heart infusion broth) and placed in a 37°C incubator for 48 hours. To evaluate the microorganisms in BHI culture medium, turbidity was assessed with a spectrophotometer that measured optical density (OD/100) (at a wavelength of 540nm).The optical density of culture medium is directly related to the total of existing bacterial in the medium. Subculture of blood agar solid medium was used to identify the target microorganism and prove the existence of E. faecalis; hence, 0.01mL was pulled out of the liquid culture medium with a sterile loop and subcultured. The morphology of the colonies of E. faecalis was evaluated both macro and microscopically (gram staining) after 24 hours. All data were statistically analysed to verify normal distribution; results were then analysed using Student t-test. The level of significance was set at P=0.05.

## RESULTS

According to kolmogrov-smirnov test, all data exhibited normal distribution. The absence of microorganism growth in the negative control group proved the aseptic and sterile working condition. Growth of microorganisms in the positive control group indicated bacterial existence within the incubation periods. Therefore, uncertainty regarding any unwanted disturbing factors during the experiment could be eliminated.

There was no significant difference between Bioglass and calcium hydroxide after 1 hour; however, after 24, 48 and 72 hours intervals calcium hydroxide showed superior antimicrobial properties (P<0.05). Antimicrobial effect of both materials increased with longer time intervals, both groups displayed significant decrease of optical density (OD/100) after 72 hours (Table 1).

**Table 1:** Optical Density (Mean±SD) of studied materials at different time intervals (hour)

**Time **	**Bioglass**	**Calcium Hydroxide**	**P value**
**1 **	131.8±0.84	125.8±6.83	0.46
**24 **	129.64±1.3	120.68±6.67	0.004
**48 **	126.36±3.61	113.96±3.67	0.000
**72 **	123.52±6.21	108.10±5.25	0.000

## DISCUSSION

Many in vitro studies have been carried out on the antimicrobial efficacy of different materials [10][11][18][19][21][22].The efficiency of direct exposure test (DET) has been previously established [19][23]. E. faecalis, a facultative anaerobe, is one of the most resistant microorganisms against disinfecting medicaments in endodontics, and is often present in resistant apical periodontitis. Therefore evaluation of antimicrobial properties of different materials against this microorganism appears reasonable [2][4][10][19][20][24]. The current study revealed that calcium hydroxide paste mixed with distilled water has greater antimicrobial efficacy against E. faecalis, compared with Bioglass after one day of exposure. Calcium hydroxide, however, could not thoroughly eliminate target microorganisms, concurring with many other studies [4][10][11][19][25]. In a recent agar diffusion experimental study, Ballal and Kundabala demonstrated calcium hydroxide paste to have weaker antimicrobial effect on Candida Albicans and E. faecalis compared to 2% CHX gel [10].

It has been shown that the efficacy of Bioglass 45S5 increases after the first 24 hours [17]; this is in agreement with our results.

Previous studies revealed more efficient results for antimicrobial effect of Bioglass S53P4 compared to Bioglass 45S5 [4][18][19]. Zehnder et al. were the first to evaluate antimicrobial effects of Bioglass S53P4 compared to calcium hydroxide paste in dentinal tubules contaminated with E. faecalis. Calcium hydroxide paste appeared completely ineffective and Bioglass suspension was able to reduce infection of the samples after five days [19]. In this study, the DET method revealed antimicrobial properties of Bioglass against resistant Enterococci, though CaOH was also effective. Krithikadatta et al. showed that the antimicrobial properties of Bioglass S53P4 and calcium hydroxide paste are similar [4]; our study was only able to demonstrate this in the first 24 hours. These inconsistencies might be due to the difference in particle proportion and percentage, the type of tested Bioglass and also the used antibacterial test. In fact, antimicrobial effect of calcium hydroxide is affected by the buffering effect of dentin, although Bioglass may be less sensitive [19].

A recent study reported that antiseptic effect of bioactive glass (BAG) S53P4 powder was stronger than that of calcium hydroxide. The formulation of Bioglass 45S5 is: Sio2 (45%), Na2o (24.5%), CaO (24.5%), P2O6 (6%), whereas, Bioglass S53P4 is Sio2 (53%), Na2o (23%), CaO (20%), P2O4 (4%).

This effect was apparently not exclusively pH-related. The presence of dentin powder in suspension increased the efficacy of S53P4 against a multiplicity of oral microorganisms [26]. The size of Bioglass particles affects antimicrobial property. The smaller the particle, the greater the exposed surface to the liquid. This results in stronger antimicrobial properties [17][18][19][20]. Commercial brands of Bioglass 45S5 composes of 90-710 micro particles. The new nanometric Bioglass has 20-60 particles which contribute to stronger antimicrobial effects [20]. Apparently, with increasing contact surface between smaller particles of nanometric glass, more alkaline material is released into the liquid medium which induces more antimicrobial effect. Basically, bioactive glasses have calcium oxide, sodium phosphor and silicon oxides in specific proportions which are responsible for the particular characteristics of the material and its strong bond to bone [12][13][14][15][16][17][18].Their high tissue compatibility makes them the most appropriate material as a bone substitute in reconstructive surgeries [12][27]. The antimicrobial effects of bioactive glass could be justified by the existence of Ca, Si, Na PO4 ions, cations in liquid medium and their release from glass which contribute to an increase of pH and osmotic pressure [17][18].The activity of released ions and the increase of pH and osmotic pressure could lead to death of microorganisms following exposure to the glass [18]. However, the accurate antimicrobial mechanism is not clear yet. Some researchers do not relate the antimicrobial activity of this material to pH as the buffering characteristics of dentine does not seem to decrease antimicrobial effect of Bioglass [19][21][22]. On the other hand, human dentin powder has increasing effects on the antimicrobial properties of Bioglass S53P4 against E. faecalis [21]. It is hypothesized that the degradation of Bioglass particles, particularly SiO2 is increased when the material is mixed with dentin powder and hence with the release of silica in the environment and an increase of antimicrobial activity is observed [21]. It is also reported that bone powder could also enhance this characteristics of Bioglass [22].

Antimicrobial characteristics of Ca(OH)2 is due to the release of OH ions into the environment, the increase of pH and therefore destruction of the microorganism cell wall [11][17][28] along with absorption of CO2 from the anaerobic environment [29][30]. Apparently, Ca(OH)2 mechanism could be pH-dependent so that it was inefficient against resistant microorganisms such as E. faecalis that are themselves resistant to high pH . The buffering property of dentine can also neutralize the alkalinity effect of calcium hydroxide when it comes to disinfect contaminated dentinal tubules [11].

A recent study, has reported that the combined effect of dentin powder and BAG is neither a matter of osmolarity or even cellular agglutination, nor is it linked to binding of bacteria to dentin powder in the presence of BAG. It was proved that only combinations of solid BAG and dentin powder and not their supernatants in suspension have an additive effect. Dentin powder with its complex surface acted as a recipient for ions in solution, and hence acted as a catalyst for the dissolution of the glass in aqueous suspension. It was this ionic flow between the glass and dentin powder that appeared to interfere with bacterial viability [26]. It should be stated that the antibacterial effect of BAGs is related to their particle size. The lesser effect of the BAG suspension, despite the fact that its initial pH is comparable to that of the Ca(OH)2 counterpart, can be explained by the lesser alkaline capacity of the glass suspension compared to Ca(OH)2. This factor needs to be considered before extrapolating the findings of the study to an in vivo situation. The in vitro study of Prabhakar and Kumar, BAG was relatively effective against E. faecalis and the addition of enamel powder did not significantly boosts its antimicrobial efficacy at the end of 24 hours and three days. However, at the end of five days, this combination exhibited marked increase in antimicrobial activity. The mixture of BAG plus dentin powder increased the antimicrobial efficacy of BAG at the end of 24 hours and three days, which was statistically significant; although it tapered at the end of five days [26].

## CONCLUSION

The current study demonstrated that the antimicrobial effect of Bioglass 45S5 against E. faecalis was lower than Ca(OH)2, although it tends to increase with time. Considering all desirable properties of Bioglass, the material could well be incorporated into endodontics practise as an intracanal medicament in the future. Further research is recommended to find more effective formulations of Bioglass.
